# Mr. Dennet, on Innoculation

**Published:** 1803-10-01

**Authors:** C. Dennett

**Affiliations:** Soho-Square


### *To the Editors of the Medical and Physical Journal.*

Gentlemen,

YOUR attention to my former papers, encourages me to solicit a place in the Medical Journal for the following Observations, as I think it of importance to the public to state, (in reply to that part of Mr. King's letter, wherein he says, the disease from my mode of inoculating, is more violent than by puncture) that I have never experienced a *single* instance of severe inflammation, but the contrary; and on that very account prefer using the quill, even when I have recent matter.

Within the last fortnight I was requested to visit a child in this neighbourhood, who had been inoculated at the Institution in Salisbury Court, by puncture; I understood the inflammation had been very great, but I was called in to examine the glands of the axilla, which were much enlarged in consequence; such a circumstance I had never before seen from vaccine inoculation. With many, I fear, I may be thought too partial to my own mode, but trust it has arisen from the best of all conviction, that of *experience*; not only in this, but the variolous also; and I hope it will not be thought irrelevant to the present subject, if I mention that this was *one* particular of my plan formerly alluded to, as I will state what gave rise to my practice.

In the beginning of the year 1786, great numbers failed with the natural small pox at Brighton, and a meeting of the inhabitants was called in consequence, to consider the propriety of general inoculation. At this meeting my father was requested to attend, and after the resolution being formed of carrying it into effect, he sent in the night an express for me, (sixteen miles) to desire I would bring all the preserved matter by me, and also to charge as many quills as I could, and bring with me by day-light; this I did, to the amount, as I should suppose, of about sixty. Two patients, with good distinct eruption, were put into a chaise and sent from our receiving house after me. As soon as I arrived at Brighton, I began inoculating some particular families we were well acquainted with, until all the matter thus taken was used; the rest of our patients, amounting in the whole to 725, were inoculated from the two patients above alluded to. The names being all taken down as inoculated, it was impossible I should be mistaken as to those who had the dry matter used; and when the time of eruption arrived, I could but remark the general mildness of the *first* inoculated, compared with the others; this, and some other particulars I then noticed, but conceived it might have arisen from the hurry of inoculating, by inserting the matter too deep, and using too much; or from the blood being heated by standing for a long time in the room, which was crowded all day; future experience explained the real fact, but I believe most of your readers will excuse any farther particulars on this head. I shall leave the physiologist to explain how this may happen, but have always considered that variolous matter, when inserted by puncture and in the fluid state, to partake more of the natural infection. I agree perfectly with Mr. Ring as to the impossibility of communicating diseases with the blood, but at the same time we meet with so many parents (and those well informed) who do believe it, and even insist on seeing the person the matter is taken from, that I never use the quill for more than one, though two or three might be inoculated when well charged.

The Report of Mr. Ricards, in the last number, gives me an opportunity of bearing testimony to his statement of the very beneficial effects from the fresh powder of sabine in syphilitic warts. I generally use it mixed in saturnine ointment; I have also made a tincture of it in aq. ammon. pura, and found it to answer better than any other application for the common warts of the hands.

I am, &c.

*Soho-Square,*
*August* 14, 1803.

C. DENNETT.

OBSERVATIONS

